# Correlation of aqueous and plasma levels of phosphorylated tau181 with age and Montreal Cognitive Assessment Scores in individuals undergoing cataract surgery

**DOI:** 10.1002/alz.087027

**Published:** 2025-01-09

**Authors:** Hemal Patel, Cason B Robbins, C. Ellis Wisely, Justin Ma, Pali P Singh, Pratap Challa, Terry Kim, Dilraj S Grewal, Sharon Fekrat

**Affiliations:** ^1^ Duke University, Durham, NC USA; ^2^ Duke Eye Center, Durham, NC USA; ^3^ Duke Department of Neurology, Durham, NC USA

## Abstract

**Background:**

Phosphorylated tau (p‐tau) 181 was isolated in aqueous humor and was correlated with other measures that might be consistent with Alzheimer’s disease (AD) including higher plasma p‐tau181 concentrations and lower Montreal Cognitive Assessment (MoCA blind version 7.1) scores.

**Method:**

Blood samples, aqueous humor samples, and MoCA scores were collected from patients at the time of cataract surgery. Plasma p‐tau181 concentrations and aqueous p‐tau181 concentrations were then determined using ultra‐sensitive single‐molecule assay ELISA (SiMoA) technology. Associations were tested between aqueous and plasma concentration levels, aqueous concentration levels and MoCA score, plasma concentration levels and MoCA score, aqueous concentration and age, and plasma concentration and age.

**Result:**

49 eyes of 49 participants were enrolled. In this cohort, average age was 71.2 years, average MoCA score was 19.4/22, average plasma p‐tau181 concentration was 8.3 pg/mL and average aqueous p‐tau181 concentration was 3.2 pg/mL. Plasma p‐tau181 (Spearman’s rank rho=0.457, p= 0.01) and aqueous p‐tau181 (Spearman’s rank rho=0.324, p= 0.048) significantly increased with age. Higher plasma p‐tau181 was significantly associated with higher aqueous p‐tau181 (rho=0.457, p= 0.015). In 43 patients with no history of cognitive impairment, plasma p‐tau181 was negatively but not significantly associated with MoCA scores (rho= ‐0.391, p= 0.065). However, aqueous p‐tau181 was significantly negatively associated with MoCA scores (rho= ‐0.556, p= 0.009) in these patients.

**Conclusion:**

p‐tau181 is measurable in aqueous humor and plasma. Its presence in the aqueous is correlated with its presence in plasma, with increasing age, and is negatively correlated with MoCA scores. Further study in individuals with mild cognitive impairment or AD confirmed by cerebrospinal fluid and volumetric MRI metrics may yield further insight.

